# Gene Expression Profiling of the Paracrine Effects of Uterine Natural Killer Cells on Human Endometrial Epithelial Cells

**DOI:** 10.1155/2014/393707

**Published:** 2014-03-26

**Authors:** Xin Gong, Zhenzhen Chen, Yanxia Liu, Qiudan Lu, Zhe Jin

**Affiliations:** ^1^Reproductive Endocrinology Centre, Dongfang Hospital of Beijing University of Chinese Medicine, No. 6 Fangxingyuan 1 Qu, Fengtai District, Beijing 100078, China; ^2^School of Chinese Materia Medica, Beijing University of Chinese Medicine, No. 6 Zhonghuan South Road, Wangjing, Chaoyang District, Beijing 100102, China

## Abstract

The endometrium contains a population of immune cells that undergo changes during implantation and pregnancy. The majority of these cells are uterine natural killer (uNK) cells; however, it is unclear how these cells interact with endometrial epithelial cells. Therefore, we investigated the paracrine effects of the uNK cell-secretion medium on the gene expression profile of endometrial epithelial cells *in vitro* through microarray analysis. Our results, which were verified by qRT-PCR and western blot, revealed that soluble factors from uNK cells alter the gene expression profiles of epithelial cells. The upregulated genes included interleukin-15 (IL-15) and interleukin-15 receptor alpha (IL-15RA), which result in a loop that stimulates uNK cell proliferation. In addition, vascular endothelial growth factor C (VEGF-C) and chemokine (C-X-C motif) ligand 10 (CXCL-10) were also determined to be upregulated in epithelial cells, which suggests that uNK cells work synergistically with epithelial cells to support implantation and pregnancy. In addition, oriental herbal medicines have been used to treat infertility since ancient times; however, we failed to find that Zi Dan Yin can regulate these endometrial paracrine effects.

## 1. Introduction

In the human endometrium, uterine leucocytes undergo cyclic changes during the menstrual cycle. Uterine adaptation to pregnancy includes highly regulated immune cell trafficking [1]. According to recent studies, uterine natural killer (uNK) cells are an* in vivo* factor that contributes to the pathological elongation of the window of endometrial receptivity [2]. The genetic deficiency of uNK cells results in a lack of remodeling of the uterine vasculature, leading to hypertrophied vascular media, swollen endothelial cells, and narrow vessel lumens [3]. Abnormal activation and numbers of uNK cells have been implicated in pregnancy complications [4].

uNK cells are the most abundant leucocytes during the implantation window and early pregnancy [5]. This sharp increase is likely due to the proliferation of existing uNK cells [6–8]. Hormonal effects may control this proliferation. However, to date, progesterone receptors have not been localized on these cells [9]; therefore, it has been proposed that progesterone may exert its effects on uNK cells indirectly via cytokines, such as interleukin-15 (IL-15), and other soluble factors [8, 10]. A previous study indicated that the paracrine communication between uterine leucocytes (>85% uNK) and uterine stromal cells can upregulate IL-15 and interleukin-15 receptor alpha (IL-15RA) to induce the proliferation of uNK cells and contributes to the support of trophoblast migration during implantation [11].

uNK cells are resident throughout the endometrium at the time of conception and throughout early pregnancy. Although uNK cells predominantly localize to the uterine stroma and the surrounding areas of the spiral arteries, Matrigel-supported cocultures of endothelial cells and uNK cells have shown that vascular endothelial growth factor C (VEGFC) producing uNK cells induce transporter 1 (TAP-1) expression in endothelial cells [12]. Furthermore, the endometrium undergoes precisely defined morphological and biochemical changes during implantation and early pregnancy; thus, there is no doubt that the preparation of epithelial cells is directed towards these process. We hypothesize that there similar paracrine communications occur between uNK cells and uterine epithelial cells. Our results revealed that soluble factors from uNK cells have substantial effects on endometrial epithelial gene expression. uNK cells were found to upregulate transcript levels in epithelial cells that are known to promote uNK cell proliferation, and these upregulated transcripts may contribute to the preparation for implantation and trophoblast migration and invasion.

In China, oriental herbal medicines have been used to treat infertility since ancient times [13]. Zi Dan Yin (ZDY), a compound of nine herbs (Table 1), was found to improve the morphology of the endometrium in mice [14]. Although the treatment effects of ZDY on mice have been confirmed, it is unclear whether ZDY contributes to the local immune responses. However, our results do not support the hypothesis that ZDY can improve the paracrine effects between uNK and endometrial epithelial cells.

## 2. Materials and Methods

### 2.1. Ethics Statement

All of the subjects understood and signed the informed consent form before participation. The experimental protocols were approved by the Ethics Committee of the Dongfang Hospital Human Ethics Committee (no. 2011090201).

### 2.2. Collection of Material

Decidual tissues were obtained from healthy women undergoing elective termination of a normal pregnancy between 7 and 8 weeks of gestation. The endometrial tissues were collected from endometrial biopsies taken during the proliferative phase of the cycle from women undergoing laparoscopy (Dongfang Hospital of Beijing University of Chinese Medicine, China) for benign disease. The average age was 30 ± 1.3 years, and the body mass index (BMI) was 22.1 ± 0.8 kg/m^2^. All of the nine women were Chinese of Han ethnicity. The exclusion criteria were hormonal stimulation, cancerous lesions, and irregular menstrual bleeding.

### 2.3. Isolation of the Uterine NK (uNK) Cells

The uNK cells were isolated as previously described [15, 16]. Briefly, the decidual tissues were extensively washed with Ca^2+^- and Mg2^+^-free Hank's balanced salt solution (HBSS) containing antibiotics, minced thoroughly between two scalpels into fragments of 1-2 mm^3^, and digested for 1 h at 37°C with gentle agitation in HBSS with 0.1% (w/v) collagenase I (Gibco, USA). The cell suspensions were layered over Ficoll-Hypaque medium (General Electric, USA) and centrifuged at 800 ×g and room temperature for 25 min. The cells at the interface were washed twice with Roswell Park Memorial Institute (RPMI)-1640 media with 10% fetal calf serum (FCS) and antibiotics. After incubation for 20 min at 4°C with anti-CD56 microbeads (Miltenyi Biotec, Ltd., Germany), the cells were washed with washing buffer (PBS, 2 mM EDTA, and 0.5%BSA (w/v)) and then loaded onto a MiniMACS Separator (MS) column in a MiniMACS magnet (Miltenyi Biotec, Ltd., Germany). After flushing the MS column three times, the CD56^+^ cells were flushed as indicated by the manufacturer. The purity of the uNK cells was >90% CD56^+^CD3^−^ according to the flow cytometric analysis. The uNK cells were cultured in RPMI 1640 media with 1% FCS and IL-15 (10 ng/mL) (R&D systems, USA), in which to maintain the viability of the purified uNK cells [17].

### 2.4. Production of uNK Cell-Secretion Medium

The uNK cell-secretion medium was prepared using 200 *μ*L of RPMI 1640 media with 1% FCS and IL-15 (10 ng/mL) containing 5 × 10^5^ of the purified uNK cells. The cell suspension was placed in the upper chamber of a 0.4 *μ*m pore hanging cell culture insert (Millipore, USA) in a 24-well tissue plate, and 1300 *μ*L of the same media without cells was placed in the lower chamber. As a result, only the soluble molecules from the uNK cells can pass through the filter into the lower chamber. The control medium was 1500 *μ*L of the RPMI 1640 media with 1% FCS and IL-15 (10 ng/mL), and this control medium was also used in the subsequent experiments. After incubation for 24 h at 37°C, the uNK cell-secretion medium (in the lower chamber) and the control medium were collected and frozen at −80°C. The cells in the upper chamber were collected, and the cell viability was measured using a live/dead viability kit (Invitrogen, USA). Only the conditional medium from uNK populations with less than 35% dead cells after overnight incubation was used in the subsequent experiments.

### 2.5. Human Endometrial Epithelial Cell Culture

Human endometrial tissue was cut using scissors and dissociated into single-cell suspensions using 0.1% (w/v) collagenase I for 50–60 min. Every 15 min, the digests were pipetted, and their dissociation was monitored by microscopy. The cell suspensions were filtered using a 40-mesh screen to separate the single cells from undigested tissue. To remove the erythrocytes, the cells were resuspended in 4 mL of Dulbecco's modified Eagle's medium and Ham's nutrient F12 (DMEM/F12) with 1% FCS, layered over Ficoll-Paque PLUS, and centrifuged for 25 min at 800 ×g. The leukocytes were removed with CD45-coated Dynabeads (Dynal Biotech, USA). The purified epithelial cell suspensions were then obtained through a further round of magnetic bead sorting using Collection Epithelial Dynabeads (Dynal Biotech, USA). The epithelial cell preparations were >95% pure.

The endometrial epithelial cells were cultured in serum-free bronchial epithelial growth medium (BEGM, Clonetics Crop) containing 2 mL of bovine pituitary extract (BPE), 0.5 mL of insulin, 0.5 mL of hydrocortisone (HC), 0.5 mL of gentamicin sulfate and amphotericin-B (GA-1000), 0.5 mL of retinoic acid, 0.5 mL of transferrin, 0.5 mL of triiodothyronine, 0.5 mL of epinephrine, and 0.5 mL of recombinant human epidermal growth factor (hEGF) (all supplied by Clonetics Corp., USA). The media was changed every five days. After two weeks, the epithelial cells were passed from six-well plates into 25 cm^2^ cell culture flasks.

### 2.6. ZDY Preparation

All of the crude drugs of ZDY were obtained from the Pharmacy Department of Dongfang Hospital of Beijing University of Chinese Medicine (Beijing Province, China). The quality of the raw herbs was controlled according to the requirement of the Pharmacopoeia of China. The aqueous extract of ZDY was prepared as follows. In brief, nine medicinal materials were mixed at the appropriate proportion, macerated for 1 h with eight volumes of distilled water and then decocted for 2 h. The cooled extract was then filtered. This extraction procedure was repeated twice. The extracts were then combined and concentrated by boiling to a final volume of 100 mL (10.3 g/mL). The ZDY extract concentration of 2 mg/mL was further diluted with the epithelial cell culture medium and passed through 0.2 *μ*m sterilization filters for use in subsequent studies. The ZDY extract was stored at 2–8°C until further use. The concentration of ZDY was used according to previous analyses using a cell counting kit-8 (CCK-8).

### 2.7. Treatment of Epithelial Cells with uNK Cell-Secretion Medium

The endometrial epithelial cells were allocated into three groups: control, uNK, and ZDY groups. The cells in the control and uNK groups were treated with DMEM medium with 10% FBS for 24 h, and the cells in the ZDY group were treated with 2 mg/mL ZDY for 24 h. All of the cells were then washed twice with PBS and placed in serum-free DMEM for 24 h prior to the subsequent experiments. The uNK cell-secretion medium was pooled from different batches to decrease the variability. The DMEM in the ZDY and uNK groups was then replaced with 80% uNK cell-secretion medium and 20% serum-free DMEM. The control group was treated with 80% control medium and 20% serum-free DMEM. After 6 h of incubation, the cells from the three groups were collected.

### 2.8. Microarray Experiments

The total RNA from endometrial epithelial cells from the control, uNK, and ZDY groups was extracted using Trizol (Invitrogen, USA). The RNA was purified using the RNeasy Mini Kit (Qiagen, Germany) according to the manufacturer's instructions, quantified by spectrophotometry. The microarray analysis was performed using the GeneChip 3′ IVT Express Kit (Affymetrix). Briefly, the total RNA was subjected to reverse transcription, first-strand cDNA synthesis, double-strand DNA,* in vitro* transcription, and fragmentation. The samples were hybridized onto the GeneChip PrimeView Human Gene Expression Array (Affymetrix, USA), which covers more than 36,000 transcripts and variants. After 16 h of hybridization at 45°C, the arrays were washed on a Fluidics Station 450 (Affymetrix, USA) and then scanned with a Scanner 3000 (Affymetrix, USA) to obtain the quantitative gene expression levels. The endometrial epithelial cells from the control, uNK, and ZDY groups were processed simultaneously throughout the experimental process, and three chips were used for each group.

### 2.9. Quantitative Real-Time PCR (qRT-PCR) Analysis

To verify the results obtained from the microarray experiments, qRT-PCR verification was performed for five genes: chemokine (C-X-C motif) ligand 10 (CXCL-10), interleukin-15 (IL-15), intercellular adhesion molecule 1 (ICAM-1), NLR family CARD domain containing 5 (NLRC-5), and interferon regulatory factor 1 (IRF-1). The cells from the control, uNK, and ZDY groups were washed twice with PBS, and the total RNA was then extracted using Trizol. The RNA was quantified by spectrophotometry. Reverse transcription was performed with 8 *μ*L of total RNA in a 20 µL reaction volume using a standard cDNA Synthesis Kit (Takara BIO, Japan). The qRT-PCR primer sequences for the target genes were self-designed, and the PCR primers were ordered from Invitrogen. The primer sequences for target genes are shown in Table 2.

For each qRT-PCR reaction, the typical thermal cycling conditions included an initial activation step of 95°C for 5 min and 45 cycles of 95°C each for 30 sec, 65°C for 35 sec, and 72°C for 5 min. The PCR reactions were performed on an ABI Prism 7700 Sequence Detection System (Applied Biosystems, USA). For comparison of the target mRNA levels, the cDNA concentration was normalized with that of the glyceraldehyde-3-phosphate dehydrogenase (GAPDH) PCR product. The target mRNA expression was analyzed using the 2-ΔΔCt algorithm.

### 2.10. Western Blot Analysis of ICAM-1

The cells from the control, uNK, and ZDY groups were lysed with RIPA lysis buffer (Applygen, China) supplemented with a protease inhibitor cocktail (Applygen, China). The protein concentrations were quantified using bicinchoninic acid (BCA; Applygen, China). The proteins were separated using 10% sodium dodecyl sulfate-polyacrylamide gel electrophoresis (SDS-PAGE) and transferred to nitrocellulose. The blots were blocked overnight in Tris-buffered-saline with Tween (TBST) containing 5% dried milk, washed three times for 5 min each in TBST, and then incubated overnight in a rocker at 4°C with a cocktail containing ICAM-1 (Abcam, UK; at a dilution of 1 : 1000) and 2% dried milk in TBST. The blots were then washed three times for 10 min each in TBST and incubated with the secondary antibody (Applygen, China; at a dilution of 1 : 10000) for 1 h at room temperature in a rocker. The blots were washed three times for 10 min each in TBST and then visualized with the Super ECL Plus Detection Reagent (Applygen, China). The ECL signals were detected with the Quantity One software (Bio-Rad, USA). GAPDH (Abcam, UK) was used as the internal control to validate the amount of protein.

### 2.11. Statistical Analysis

All of the variables were tested in three different culture experiments. The data were analyzed by ANOVA. In the microarray experiments, the significance level was determined by ANOVA with Benjamini-Hochberg correction (*P* < 0.001). We selected the up- and downregulated genes that showed a median fold change greater than 2 and a *P* < 0.001. The data from the qRT-PCR and western blot analyses are presented as means ± SEM after normalization with the average value of the housekeeping gene obtained for each group, and the significance level was *P* < 0.01. The graphs of the data were produced using the Microsoft Excel software.

## 3. Results

### 3.1. Microarray Experiments

Gene expression profiling using a microarray was used to compare the transcript expression levels in epithelial cells treated with control medium, uNK cell-secretion medium, and ZDY+ uNK cell-secretion medium.

This analysis identified 169 transcripts that exhibited a statistically significant change in their median expression level (either upregulated or downregulated) in response to uNK cell-secretion medium compared with their control level. These transcripts are listed in Table 3. A total of 40 genes were found upregulated, and 129 genes were downregulated. The largest group of upregulated genes included immunomodulatory gene regulators, cytokines, such as interleukin-15 (IL-15), chemokine (C-X-C motif) ligand 10 (CXCL-10), and vascular endothelial growth factor C (VEGF-C), and cytokine receptors, such as interleukin-15 receptor alpha (IL-15RA). Additionally, the upregulated transcripts encode proteins associated with transport, structuration, and transcription. The level of adhesion molecule intercellular adhesion molecule 1 (ICAM-1) was also altered. ICAM-1 binds to the leucocyte integrins LFA-1 and Mac-1 [18, 19], which are expressed on uNK cells. Thus, this change in the level of ICAM-1 should increase the localized interactions between uNK cells and epithelial cells. A number of transcripts regulating enzyme activity, such as the deltex 3-like (Drosophila) (DYX3L) and the proteasome (prosome, macropain) subunit beta type 9 (large multifunctional peptidase 2) (PSMB9), and several transcripts regulating nucleotide metabolism genes, such as guanylate binding protein 5 (GBP5) and guanylate binding protein 1 interferon-inducible (GBP1), were also upregulated. In addition, genes with various functions, such as the icon binding complement component 1S subcomponent (C1S), SP110 nuclear body protein (SP110), tryptophan metabolism indoleamine 2,3-dioxygenase 1 (IDO1), and tryptophanyl-tRNA synthetase (WARS), and several genes with unknown function were found to be upregulated. The largest group of downregulated genes encodes transcription factors, such as B double prime 1 subunit of RNA polymerase III transcription initiation factor IIIB (BDP1) and thyroid hormone receptor interactor 11 (TRIP11). The levels of immunomodulatory gene regulators, including interleukin-36 gamma (IL-36G) and interleukin-33 (IL-33), were also altered. The levels of transcripts encoding molecules responsible for signaling, transport, structuration, and cell-cell adhesion were also downregulated. In addition, genes with various functions, such as ion binding, kinase, enzyme activity, and nucleotide metabolism, were found to be downregulated.

The microarray analysis identified 51 transcripts in epithelial cells that exhibited a statistically significant change in their median expression level (either upregulated or downregulated) in response to pretreatment with ZDY and treatment with the uNK cell-secretion medium compared with their level in response to treatment with uNK cell-secretion medium (Table 4). Of these genes, 33 were downregulated, and 18 were upregulated. The largest group of upregulated genes includes the following: enzyme asparagine synthetase (glutamine-hydrolyzing) (ASNS) and aldo-keto reductase family 1 member C1 (dihydrodiol dehydrogenase 1; 20-alpha (3-alpha)-hydroxysteroid dehydrogenase) (AKR1C1). The transcripts encoding molecules responsible for signal transduction, namely, aldo-keto reductase family 1 member C2 (dihydrodiol dehydrogenase 2; bile acid binding protein; 3-alpha hydroxysteroid dehydrogenase, type III) (AKR1C2) and structuration, such as talin 1 (TLN 1) and keratin 75 (KRT75), were also upregulated. The levels of transcripts encoding proteins that regulate transcription and ion binding, such as nuclear protein transcriptional regulator 1 (NUPR1) and S100 calcium binding protein A8 (S100A8), were also altered, and several genes with unknown function were found to be upregulated. The downregulated transcripts included only two genes of known function that were downregulated by more than 4fold: lamin B1 (LMNB1, a structural factor) and tankyrase, TRF1-interacting ankyrin-related ADP-ribose polymerase 2 (TNKS2, an enzyme activator). The genes that were downregulated by more than 2fold included the genes encoding the cytokines chemokine (C-X-C motif) ligand 3 (CXCL3) and caprin family member 2 (CAPRIN2), the transporter component of the oligomeric golgi complex 5 (COG5), and various genes associated with transcription, kinase, ion binding, and nucleotide metabolism.

### 3.2. Quantitative Real-Time RT-PCR (qRT-PCR)

**Figure 1:**
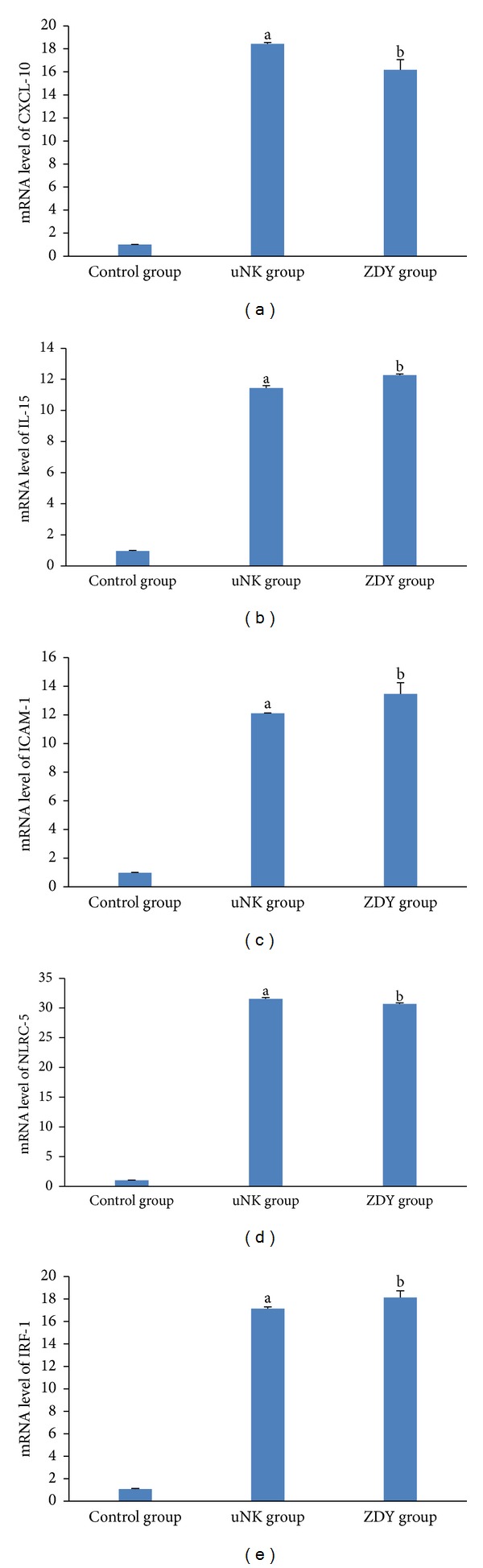
Transcription levels of (a) CXCL-10, (b) IL-15, (c) ICAM-1, (d) NLRC-5, and (e) IRF-1 determined by qRT-PCR. The relative fold changes are shown in Section 3. The qRT-PCR results were consistent with the microarray analysis. The data are expressed as means ± SEM. ^a^ *P* < 0.001 for the comparison between the uNK and control groups; ^b^ *P* < 0.001 for the comparison between the ZDY and control groups.

To verify these changes, the transcript levels of the following genes were measured by qRT-PCR: CXCL-10, IL-15, ICAM-1, NLRC-5, and IRF-1 (Figure 1). The qRT-PCR results confirmed the substantial and significant changes in expression observed in the microarray analysis. Epithelial cells treated with the uNK cell-secretion medium showed increased expression levels of CXCL-10 (18.4-fold), IL-15 (11.7-fold), ICAM-1 (12.5-fold), NLRC-5 (31.5-fold), and IRF-1 (15.6-fold) (all *P* < 0.001) compared with the control group.

We also confirmed that epithelial cells pretreated with ZDY and then treated with the uNK cell-secretion medium exhibited downregulated expression levels of CXCL-10 (0.9-fold; 0.01 < *P* < 0.05) and NLRC-5 (1.0-fold;0.01 < *P* < 0.05) and upregulated levels of IL-15 (1.1-fold; 0.01 < *P* < 0.05), ICAM-1(1.1-fold; *P* > 0.05), and IRF-1 (1.1-fold; *P* > 0.05) compared with epithelial cells treated with the uNK cell-secretion medium. However, none of these changes were significant.

### 3.3. Western Blot

**Figure 2:**
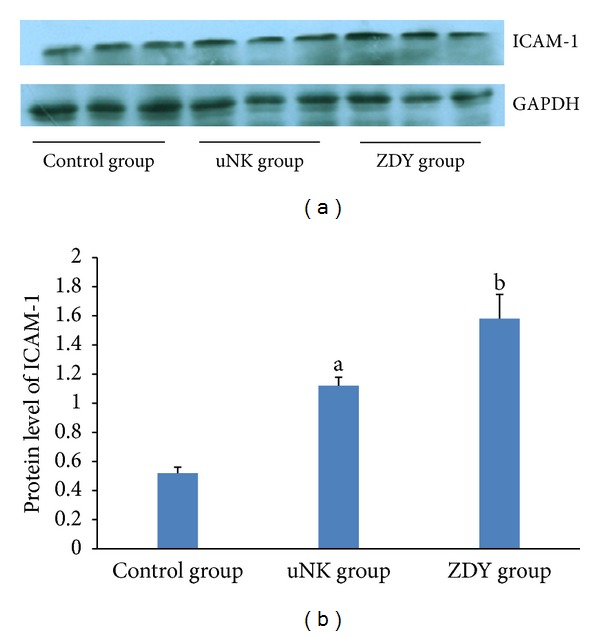
(a) Expression of endometrial ICAM-1 protein in endometrial epithelial cells. The GAPDH band was used as the internal loading control in each lane. (b) Comparison of the normalized expression intensity of endometrial ICAM-1 protein in the control, uNK, and ZDY groups. The data are expressed as means ± SEM. ^a^ *P* < 0.01 for the comparison between the uNK and control groups; ^b^ *P* < 0.01 for the comparison between the ZDY and control groups.

To confirm these effects at the protein level, the amount of ICAM-1 was analyzed by western blot (Figure 2). Epithelial cells treated with the uNK cell-secretion medium showed a 2.1-fold (*P* < 0.01) increase in the expression of ICAM-1. However, the epithelial cells treated with ZDY and the uNK cell-secretion medium showed a 1.4-fold increase (0.01 < *P* < 0.05) in the expression of ICAM-1 compared with the epithelial cells treated with the uNK cell-secretion medium. Thus, there was no significant difference between the uNK and ZDY groups.

## 4. Discussion

Progesterone plays a central role in reproduction and is involved in implantation and pregnancy. When pregnancy occurs, high progesterone levels are critical not only for facilitating implantation but also for maintaining pregnancy by stimulating uterine growth [20, 21]. Several observations suggest that uNK cells also play an important role in reproduction: (a) these cells are hormonally regulated because they increase in number during the luteal phase when implantation occurs [7], (b) these cells are present in early gestation at the time placental cells invade the maternal arteries [22], and (c) these cells are particularly abundant around trophoblast cells [23]. The proliferation of uNK cells is the foundation of their reproductive function. One of the mechanisms underlying the increase in uNK cell numbers is the stimulation of the proliferation of existing uNK cells by progesterone. In mouse uterine tissues, the significant upregulation in the decidual NK population is regulated by progesterone [24]. Because there are no progesterone receptors on uNK cells, it is hypothesized that the paracrine communication between uNK and epithelial cells participates in this process. In this study, we used the uNK cell-secretion medium to stimulate epithelial cells in an attempt to find the interaction and/or paracrine effects between these cells. The data obtained in the present study demonstrate that uNK cells have a significant effect on the gene expression in epithelial cells in the nonpregnant state, and these paracrine effects also have a profound effect on uNK proliferation.

The largest group of upregulated genes induced in epithelial cells by the soluble factors derived from uNK cells induced cytokines and immunological factors, including IL-15 and IL-15RA. IL-15 is critical for NK cell differentiation in mice and human lymphoid tissue [25, 26]. IL-15 acts on precursor and immature NK cells. In humans, IL-15 transcription is more abundant during the secretory compared with the proliferative cycle phase, is maintained in early pregnancy, and localizes to the endothelium and decidual spiral arteries [27]. The correlative time strongly suggests that IL-15 participates in uNK cell proliferation and that IL-15 may also contribute to the localization of uNK cells. Our results show that uNK cells exhibit upregulated levels of both IL-15 and IL-15RA. This may activate a powerful proliferation loop because IL-15 is presented by IL-15RA on epithelial cells to promote uNK proliferation.

There are temporal and quantitative features associated with the activities of uNK cells and trophoblast cells in uterine spiral artery maturation. There is evidence that IL-15 upregulates the level of VEGF-C mRNA expression, which suggests that uNK cells may play an important role in endometrial angiogenesis and regeneration [28]. In addition, CXCL-10 is involved in uNK cell production, directs the migration and invasion of CXCR1+, CXCR3+, CXCR4+, and CCR3+ trophoblast [12], and promotes angiogenesis in the placental bed [29]. Furthermore, in the absence of uNK cells, spiral artery extension is delayed, and the tunica media remain intact [30], and these studies are consistent with an angiogenic/vascular remodeling role for uNK cells [4, 12, 31]. We found some evidence that uNK cells promote changes in endometrial epithelial cells in the nonpregnant state. The level of VEGF-C, which is upregulated in epithelial cells by the uNK cell-secretion medium, increases markedly during implantation and early pregnancy [32]. Our data suggest that uNK may prime some of the decidualization changes that occur in epithelial cells prior to the onset of full decidualization.

**Figure 3:**
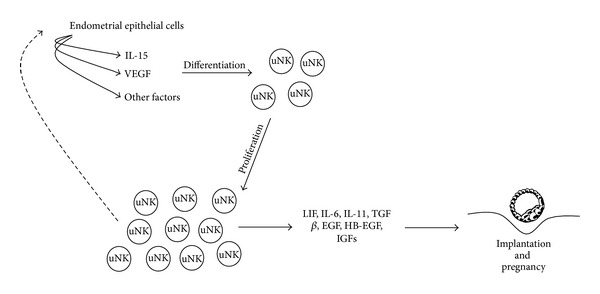
Model of the proliferation and function of uNK cells. During implantation and early pregnancy, the uNK paracrine signals stimulate endometrial epithelial cells to produce IL-15, VEGF, and other unidentified factors that may regulate uNK cell proliferation. The immune paracrine communication between uNK and endometrial epithelial cells may support implantation and trophoblast development.

The exact function of uNK cells has not yet been unequivocally determined, but it is known that these cells express a different cytokine profile compared with peripheral NK cells. For example, these cells express colony-stimulating factor (CSF), macrophage colony-stimulating factor (M-CSF), granulocyte-macrophage colony-stimulating factor (GM-CSF), tumor necrosis factor alpha (TNF-*α*), interferon-*γ* (IFN-*γ*), and leukemia inhibitory (LIF) [33, 34]. During human pregnancy, these cells are found in close proximity to the implantation site and in close contact with the infiltrating extravillous trophoblasts. Furthermore, uNK cells increase their numbers in early pregnancy due to hormonal dependence and exhibit close proximity to trophoblasts. Taken together, these data support the conclusion that uNK cells play an important role in the regulation for the maternal immune response for the control of implantation and trophoblast invasion during human pregnancy. The summary of the paracrine effects of uNK cells on the epithelial cells is shown in Figure 3.

In addition, this study provides the first demonstration of the paracrine communication between uNK and endometrial epithelial cells. Because our previous study showed that ZDY can improve the morphology of the endometrium in mice, we attempted to identify a relationship between the paracrine communication (uNK/epithelial cells) and the mechanism of action of ZDY. However, the majority of the up- and downregulated genes induced in the ZDY pretreated epithelial cells were of unknown function. To the best of our knowledge, there are no published studies on the effects of traditional Chinese medicine on the human endometrial paracrine communication. Our results suggest that ZDY may not regulate this process* in vitro*. ZDY may improve the morphology of the uterus through another mechanism.

## 5. Conclusions

Overall, this study provides the first detailed demonstration of the paracrine interaction between immune cells and epithelial cells in the endometrial environment. The findings indicate that these paracrine signals may contribute to uNK cell proliferation during implantation and early pregnancy. In addition, we failed to find evidence indicating that ZDY regulates the paracrine effects between uNK cells and epithelial cells* in vitro*.

## Supplementary Material

Hierarchical Clustering of microarray experiments. SP-ZY represented the ZDY group; SP-uNK represented the uNK group and SP-K represented the control group. Three chips were used for each group.

## Figures and Tables

**Table 1:** Composition of Zi Dan Yin (ZDY).

Components	Ratio
(1) Sheng Di (*Rehmannia glutinosa *(Gaertn.) Libosch., root)	15
(2) Dan Shen (*Salviae Miltiorrhizae *Bge., root)	10
(3) Dang gui (*Angelica sinensis *(Oliv.) Diels., root)	12
(4) Chuan Duan (*Dipsacus asperoides* C. Y. Cheng et T.M. Ai., root)	15
(5) Du Zhong (*Eucommia ulmoides* Oliv., cortex)	12
(6) Shan Yao (*Dioscorea opposita *Thunb., rhizome)	15
(7) Mei Gui-hua (*Rosa rugosa *Thunb., flower)	6
(8) Chuan Xiong (*Ligusticum Chuanxiong *Hort., rhizome)	6
(9) Yi Yi-ren (*Coix lacryma-jobi *L. var. ma-yuen (Roman.) Stapf., seed)	12

**Table 2:** Sequences of primers used in the quantitative real-time PCR analysis.

Gene	Forward primer	Reverse primer	Amplicon
CXCL-10	CTTTCTGACTCTAAGTGGCATTC	CACCCTTCTTTTTCATTGTAGCAA	176 bp
IL-15	TGGCTGCTGGAAACCC	CACAAGTAGCACTGGATGGAAAT	123 bp
ICAM-1	GAGGAAGGAGCAAGACTCAA	AGCATACCCAATAGGCAGCAAG	141 bp
NLRC-5	AAACTTGATGACTCCTCCCTTACTT	TTAGACCTGGCTTTGTCCCTTAC	120 bp
IRF-1	CCAGAAAAGCATAACACCAATCC	CCACTTTCCTTCACATTTCACTG	144 bp
GAPDH	GAGCCAAAAGGGTCATCATCT	AGGGGCCATCCACAGTCTTC	231 bp

**Table 3:** Transcripts that were altered by more than twofold in endometrial epithelial cells by stimulation with the uNK cell-secretion medium (*P* < 0.001). The transcript levels were obtained using a GeneChip PrimeView Human Gene Expression Array.

Gene symbol	Fold change	Gene ID	Description
Upregulated genes: 40 transcripts			
Cytokines/membrane proteins/immunological factors			
NLRC5	14.1	84166	NLR family, CARD domain containing 5
IL15	5.8	3600	Interleukin-15
IFIT3	5.8	3437	Interferon-induced protein with tetratricopeptide repeats 3
CXCL10	3.1	3627	Chemokine (C-X-C motif) ligand 10
IFIH1	2.9	64135	Interferon induced with helicase C domain 1
IFIT5	2.8	24138	Interferon-induced protein with tetratricopeptide repeats 5
IL-15RA	2.7	3601	Interleukin-15 receptor, alpha
VEGFC	2.5	7424	Vascular endothelial growth factor C
PDCD1LG2	2.5	80380	Programmed cell death 1 ligand 2
Transporters			
TAP1	3.4	6890	Transporter 1, ATP-binding cassette, sub-family B (MDR/TAP)
TAP2	2.6	6891	Transporter 2, ATP-binding cassette, sub-family B (MDR/TAP)
APOL3	2.1	80833	Apolipoprotein L, 3
VLDLR	2.1	7436	Very low density lipoprotein receptor
APOL2	2.1	23780	Apolipoprotein L, 2
Structural factors			
LMNB1	4.3	4001	Lamin B1
Transcription			
IRF1	11	3659	Interferon regulatory factor 1
IRF9	4.9	10379	Interferon regulatory factor 9
STAT1	4.5	6772	Signal transducer and activator of transcription 1, 91 kda
TAF15	2.1	8148	TAF15 RNA polymerase II, TATA box binding protein (TBP)-associated factor, 68 kda
Cell-cell adhesion			
ICAM1	5.1	3383	Intercellular adhesion molecule 1
Ion binding			
C1S	3.2	716	Complement component 1, s subcomponent
SP110	2.9	3431	SP110 nuclear body protein
PARP12	2.3	64761	Poly (ADP-ribose) polymerase family, member 12
ZC3HAV1	2.3	56829	Zinc finger CCCH-type, antiviral 1
Tryptophan metabolism			
IDO1	6.2	3620	Indoleamine 2,3-dioxygenase 1
WARS	4.2	7453	Tryptophanyl-trna synthetase
Enzyme activity			
DTX3L	4.6	151636	Deltex 3-like (Drosophila)
PSMB9	3.3	5698	Proteasome (prosome, macropain) subunit, beta type, 9 (large multifunctional peptidase 2)
MSRB1	2.5	51734	Methionine sulfoxide reductase B1
CTSZ	2.2	1522	Cathepsin Z
Nucleotide metabolism			
GBP5	10.5	115362	Guanylate binding protein 5
GBP1	5.8	2633	Guanylate binding protein 1, interferon-inducible
PARP14	5.2	54625	Poly (ADP-ribose) polymerase family, member 14
GBP2	3.2	2634	Guanylate binding protein 2, interferon-inducible
MX1	2.5	4599	Myxovirus (influenza virus) resistance 1, interferon-inducible protein p78 (mouse)
RAD23B	2.3	5887	RAD23 homolog B (S. Cerevisiae)
GBP7	2	388646	Guanylate binding protein 7
Others			
FAM117B	3.6	150864	Family with sequence similarity 117, member B
C19orf66	2.6	55337	Chromosome 19 open reading frame 66
NUB1	2.5	51667	Negative regulator of ubiquitin-like proteins 1

Downregulated genes: 129 transcripts			
Cytokines/membrane proteins/immunological factors			
IL36G	−3.4	56300	Interleukin-36, gamma
IL33	−3.0	90865	Interleukin-33
IL1RN	−2.5	3557	Interleukin-1 receptor antagonist
DNAJB14	−2.5	79982	Dnaj (Hsp40) homolog, subfamily B, member 14
KITLG	−2.3	4254	KIT ligand
LARP4	−2.3	113251	La ribonucleoprotein domain family, member 4
CLDN4	−2.2	1364	Claudin 4
CUL5	−2.2	8065	Cullin 5
Intracellular/signalling factors			
ITGB8	−3.5	3696	Integrin, beta 8
RICTOR	−2.8	253260	RPTOR independent companion of MTOR, complex 2
IL6ST	−2.6	3572	Interleukin-6 signal transducer (gp130, oncostatin M receptor)
SOCS4	−2.5	122809	Suppressor of cytokine signaling 4
NRIP1	−2.5	8204	Nuclear receptor interacting protein 1
SEMA3A	−2.5	10371	Sema domain, immunoglobulin domain (Ig), short basic domain, secreted, (semaphorin) 3A
PLCB4	−2.2	5332	Phospholipase C, beta 4
COPS2	−2.2	9318	COP9 constitutive photomorphogenic homolog subunit 2 (Arabidopsis)
BMPR2	−2	659	Bone morphogenetic protein receptor, type II (serine/threonine kinase)
Transporters			
TPR	−3.4	7175	Translocated promoter region, nuclear basket protein
KIAA1033	−2.5	23325	Kiaa1033
BAZ2B	−2.5	29994	Bromodomain adjacent to zinc finger domain, 2B
TMED5	−2.2	50999	Transmembrane emp24 protein transport domain containing 5
SLC4A7	−2	9497	Solute carrier family 4, sodium bicarbonate cotransporter, member 7
Structural factors			
ROCK2	−2.9	9475	Rho-associated, coiled-coil containing protein kinase 2
Transcription			
BDP1	−3.6	55814	B double prime 1, subunit of RNA polymerase III transcription initiation factor IIIB
TRIP11	−3.4	9321	Thyroid hormone receptor interactor 11
ZNF644	−3.3	84146	Zinc finger protein 644
CHD9	−3.2	80205	Chromodomain helicase DNA binding protein 9
ZNF480	−3.1	147657	Zinc finger protein 480
ZNF292	−3	23036	Zinc finger protein 292
ZNF148	−2.8	7707	Zinc finger protein 148
ZNF267	−2.8	10308	Zinc finger protein 267
ZBTB20	−2.7	26137	Zinc finger and BTB domain containing 20
CREB1	−2.7	1385	Camp responsive element binding protein 1
ZNF638	−2.6	27332	Zinc finger protein 638
ZNF518A	−2.6	9849	Zinc finger protein 518A
ESF1	−2.6	51575	ESF1, nucleolar pre-rrna processing protein, homolog (S. Cerevisiae)
FAR1	−2.6	84188	Fatty acyl coa reductase 1
ZNF91	−2.5	7644	Zinc finger protein 91
CENPF	−2.5	1063	Centromere protein F, 350/400 kda (mitosin)
FOXN2	−2.4	3344	Forkhead box N2
DNTTIP2	−2.2	30836	Deoxynucleotidyl transferase, terminal, interacting protein 2
TMF1	−2.2	7110	TATA element modulatory factor 1
BCLAF1	−2.2	9774	BCL2-associated transcription factor 1
CEBPZ	−2.2	10153	CCAAT/enhancer binding protein (C/EBP), zeta
ZNF146	−2.1	7705	Zinc finger protein 146
HIF1A	−2	3091	Hypoxia inducible factor 1, alpha subunit (basic helix-loop-helix transcription factor)
Cell-cell adhesion			
DST	−2.9	667	Dystonin
Ion binding			
ATRX	−4.6	546	Alpha thalassemia/mental retardation syndrome X-linked
EEA1	−3.4	8411	Early endosome antigen 1
RSF1	−2.6	51773	Remodeling and spacing factor 1
NIN	−2.6	51199	Ninein (GSK3B interacting protein)
SCAF11	−2.5	9169	SR-related CTD-associated factor 11
MIB1	−2	57534	Mind bomb E3 ubiquitin protein ligase 1
TRMT13	−2	54482	Trna methyltransferase 13 homolog (S. Cerevisiae)
Kinase			
CCDC88A	−3.1	55704	Coiled-coil domain containing 88A
FER	−3.1	2241	Fer (fps/fes related) tyrosine kinase
KTN1	−3	3895	Kinectin 1 (kinesin receptor)
SCYL2	−2.6	55681	SCY1-like 2 (S. Cerevisiae)
AKAP11	−2.5	11215	A kinase (PRKA) anchor protein 11
PIK3C2A	−2.5	5286	Phosphatidylinositol-4-phosphate 3-kinase, catalytic subunit type 2 alpha
CDK6	−2.3	1021	Cyclin-dependent kinase 6
NEK1	−2.3	4750	NIMA (never in mitosis gene a)-related kinase 1
SLK	−2.2	9748	STE20-like kinase
UTRN	−2.2	7402	Utrophin
RB1CC1	−2	9821	RB1-inducible coiled-coil 1
YES1	−2	7525	V-yes-1 Yamaguchi sarcoma viral oncogene homolog 1
Enzyme activity			
RANBP2	−2.9	5903	RAN binding protein 2
JMJD1C	−2.7	221037	Jumonji domain containing 1C
RNF6	−2.7	6049	Ring finger protein (C3H2C3 type) 6
LTN1	−2.6	26046	Listerin E3 ubiquitin protein ligase 1
RBBP6	−2.5	5930	Retinoblastoma binding protein 6
PPIG	−2.5	9360	Peptidylprolyl isomerase G (cyclophilin G)
PTAR1	−2.4	375743	Protein prenyltransferase alpha subunit repeat containing 1
PYROXD1	−2.3	79912	Pyridine nucleotide-disulphide oxidoreductase domain 1
LIG4	−2.3	3981	Ligase IV, DNA, ATP-dependent
RAD50	−2.1	10111	RAD50 homolog (S. Cerevisiae)
DBF4	−2	10926	DBF4 homolog (S. Cerevisiae)
Nucleotide metabolism			
CHD1	−2.6	1105	Chromodomain helicase DNA binding protein 1
CHML	−2.5	1122	Choroideremia-like (Rab escort protein 2)
ELMOD2	−2.1	255520	ELMO/CED-12 domain containing 2
NAA15	−2	80155	N(alpha)-acetyltransferase 15, nata auxiliary subunit
KIF20B	−2	9585	Kinesin family member 20B
Others			
GOLGA4	−3.9	2803	Golgin A4
ASPM	−3.4	259266	Asp (abnormal spindle) homolog, microcephaly associated (Drosophila)
CEP350	−3.3	9857	Centrosomal protein 350 kda
C10orf118	−3.2	55088	Chromosome 10 open reading frame 118
GALNT5	−3.2	11227	UDP-N-acetyl-alpha-D-galactosamine:polypeptide N-acetylgalactosaminyltransferase 5 (galnac-T5)
BOD1L1	−3.1	259282	Biorientation of chromosomes in cell division 1-like 1
ANKRD12	−3.1	23253	Ankyrin repeat domain 12
RIF1	−3	55183	RAP1 interacting factor homolog (yeast)
KIAA2026	−3	158358	Kiaa2026
FAM111B	−2.9	374393	Family with sequence similarity 111, member B
EIF5B	−2.8	9669	Eukaryotic translation initiation factor 5B
OSBPL8	−2.8	114882	Oxysterol binding protein-like 8
GOLGB1	−2.8	2804	Golgin B1
MALAT1	−2.7	378938	Metastasis associated lung adenocarcinoma transcript 1 (non-protein coding)
PRPF40A	−2.7	55660	PRP40 pre-mrna processing factor 40 homolog A (S. Cerevisiae)
QSER1	−2.6	79832	Glutamine and serine rich 1
THOC2	−2.6	57187	THO complex 2
RASSF6	−2.6	166824	Ras association (ralgds/AF-6) domain family member 6
STAG2	−2.6	10735	Stromal antigen 2
NUFIP2	−2.5	57532	Nuclear fragile X mental retardation protein interacting protein 2
SMC2	−2.5	10592	Structural maintenance of chromosomes 2
SLC30A1	−2.5	7779	Solute carrier family 30 (zinc transporter), member 1
ZNF268	−2.4	10795	Zinc finger protein 268
KIAA1109	−2.4	84162	Kiaa1109
NIPBL	−2.4	25836	Nipped-B homolog (Drosophila)
ANKRD30BP2	−2.4	149992	Ankyrin repeat domain 30B pseudogene 2
KIF5B	−2.4	3799	Kinesin family member 5B
SMC4	−2.3	10051	Structural maintenance of chromosomes 4
SMC5	−2.3	23137	Structural maintenance of chromosomes 5
NEMF	−2.3	9147	Nuclear export mediator factor
GCC2	−2.3	9648	GRIP and coiled-coil domain containing 2
EXOC5	−2.3	10640	Exocyst complex component 5
FCHO2	−2.3	115548	FCH domain only 2
SMC6	−2.3	79677	Structural maintenance of chromosomes 6
HOOK3	−2.2	84376	Hook homolog 3 (Drosophila)
LGALSL	−2.1	29094	Lectin, galactoside-binding-like
SEMA3C	−2.1	10512	Sema domain, immunoglobulin domain (Ig), short basic domain, secreted, (semaphorin) 3C
TMEM106B	−2.1	54664	Transmembrane protein 106B
PCM1	−2.1	5108	Pericentriolar material 1
KRTAP2-3	−2.1	730755	Keratin associated protein 2-3
MACC1	−2.1	346389	Metastasis associated in colon cancer 1
CCDC82	−2.1	79780	Coiled-coil domain containing 82
TTC37	−2.1	9652	Tetratricopeptide repeat domain 37
UACA	−2.1	55075	Uveal autoantigen with coiled-coil domains and ankyrin repeats
CEP290	−2	80184	Centrosomal protein 290 kda
USP53	−2	54532	Ubiquitin specific peptidase 53
METRNL	−2	284207	Meteorin, glial cell differentiation regulator-like

**Table 4:** Transcripts that were altered by more than twofold in endometrial epithelial cells by stimulation with ZDY and uNK cell-secretion medium (*P* < 0.001). The transcript levels were obtained using a GeneChip PrimeView Human Gene Expression Array.

Gene symbol	Fold change	Gene ID	Description
Upregulated genes: 18 transcripts			
Intracellular/signalling factors			
AKR1C2	2.7	1646	Aldo-keto reductase family 1, member C2 (dihydrodiol dehydrogenase 2; bile acid binding protein; 3-alpha hydroxysteroid dehydrogenase, type III)
Structural factors			
TLN1	2.7	7094	Talin 1
KRT75	2.0	9119	Keratin 75
Transcription			
NUPR1	2.6	26471	Nuclear protein, transcriptional regulator, 1
Ion binding			
S100A8	2.1	6279	S100 calcium binding protein A8
Enzyme activity			
ASNS	5.2	440	Asparagine synthetase (glutamine-hydrolyzing)
AKR1C1	3.3	1645	Aldo-keto reductase family 1, member C1 (dihydrodiol dehydrogenase 1; 20-alpha (3-alpha)-hydroxysteroid dehydrogenase)
NQO1	2.9	1728	NAD(P)H dehydrogenase, quinone 1
CBS	2.5	875	Cystathionine-beta-synthase
AKR1C3	2.3	8644	Aldo-keto reductase family 1, member C3 (3-alpha hydroxysteroid dehydrogenase, type II)
PSAT1	2.2	29968	Phosphoserine aminotransferase 1
HMOX1	2.2	3162	Heme oxygenase (decycling) 1
GPX2	2.2	2877	Glutathione peroxidase 2 (gastrointestinal)
DHRS3	2.1	9249	Dehydrogenase/reductase (SDR family) member 3
H1FX	2.1	8971	H1 histone family, member X
Others			
SLC7A11	3.1	23657	Solute carrier family 7 (anionic amino acid transporter light chain, xc-system), member 11
CTH	2.4	1491	Cystathionase (cystathionine gamma-lyase)
HSPB8	2.2	26353	Heat shock 22 kda protein 8

Downregulated genes: 33 transcripts			
Cytokines/membrane proteins/signalling factors			
CXCL3	−2.1	2921	Chemokine (C-X-C motif) ligand 3
CAPRIN2	−2.0	65981	Caprin family member 2
Transporters			
COG5	−2.0	10466	Component of oligomeric golgi complex 5
Structural factors			
LMNB1	−5.5	4001	Lamin B1
PNN	−2.9	5411	Pinin, desmosome associated protein
MFAP5	−2.2	8076	Microfibrillar associated protein 5
RPL37A	−2.2	6168	Ribosomal protein l37a
Transcription			
HIRA	−3.6	7290	HIR histone cell cycle regulation defective homolog A (S. Cerevisiae)
YAP1	−2.4	10413	Yes-associated protein 1
ZNF226	−2.4	7769	Zinc finger protein 226
TAF15	−2.1	8148	TAF15 RNA polymerase II, TATA box binding protein (TBP)-associated factor, 68 kda
NFKBIZ	−2.1	64332	Nuclear factor of kappa light polypeptide gene enhancer in B-cells inhibitor, zeta
Ion binding			
ZDHHC11	−2.2	79844	Zinc finger, DHHC-type containing 11
RNF145	−2.1	153830	Ring finger protein 145
Kinase			
AHSA2	−2.0	130872	AHA1, activator of heat shock 90 kda protein atpase homolog 2 (yeast)
Enzyme activity			
TNKS2	−7.7	80351	Tankyrase, TRF1-interacting ankyrin-related ADP-ribose polymerase 2
Nucleotide metabolism			
RAD23B	−2.0	5887	RAD23 homolog B (S. Cerevisiae)
RCC1	−2.0	1104	Regulator of chromosome condensation 1
Others			
GOLGA8A	−5.4	23015	Golgin A8 family, member A
LUC7L3	−3.8	51747	LUC7-like 3 (S. Cerevisiae)
HNRNPA2B1	−2.9	3181	Heterogeneous nuclear ribonucleoprotein A2/B1
XIST	−2.8	7503	X (inactive)-specific transcript (non-protein coding)
SNORA33	−2.6	594839	Small nucleolar RNA, H/ACA box 33
SNHG12	−2.5	85028	Small nucleolar RNA host gene 12 (non-protein coding)
C20orf197	−2.4	284756	Chromosome 20 open reading frame 197
EML2	−2.4	24139	Echinoderm microtubule associated protein like 2
PNISR	−2.3	25957	PNN-interacting serine/arginine-rich protein
KIAA0020	−2.3	9933	Kiaa0020
HMMR	−2.2	3161	Hyaluronan-mediated motility receptor (RHAMM)
NOP58	−2.2	51602	NOP58 ribonucleoprotein homolog (yeast)
PILRB	−2	29990	Paired immunoglobin-like type 2 receptor beta
NCAPG	−2	64151	Non-SMC condensin I complex, subunit G
PICALM	−2	8301	Phosphatidylinositol binding clathrin assembly protein
