# Supplementary figures and images for: Gene Expression Profiling of the Paracrine Effects of Uterine Natural Killer Cells on Human Endometrial Epithelial Cells
Source: Int J Endocrinol. 2014 Mar 26;2014:393707. doi: 10.1155/2014/393707 (PMC3984803; doi:10.1155/2014/393707)

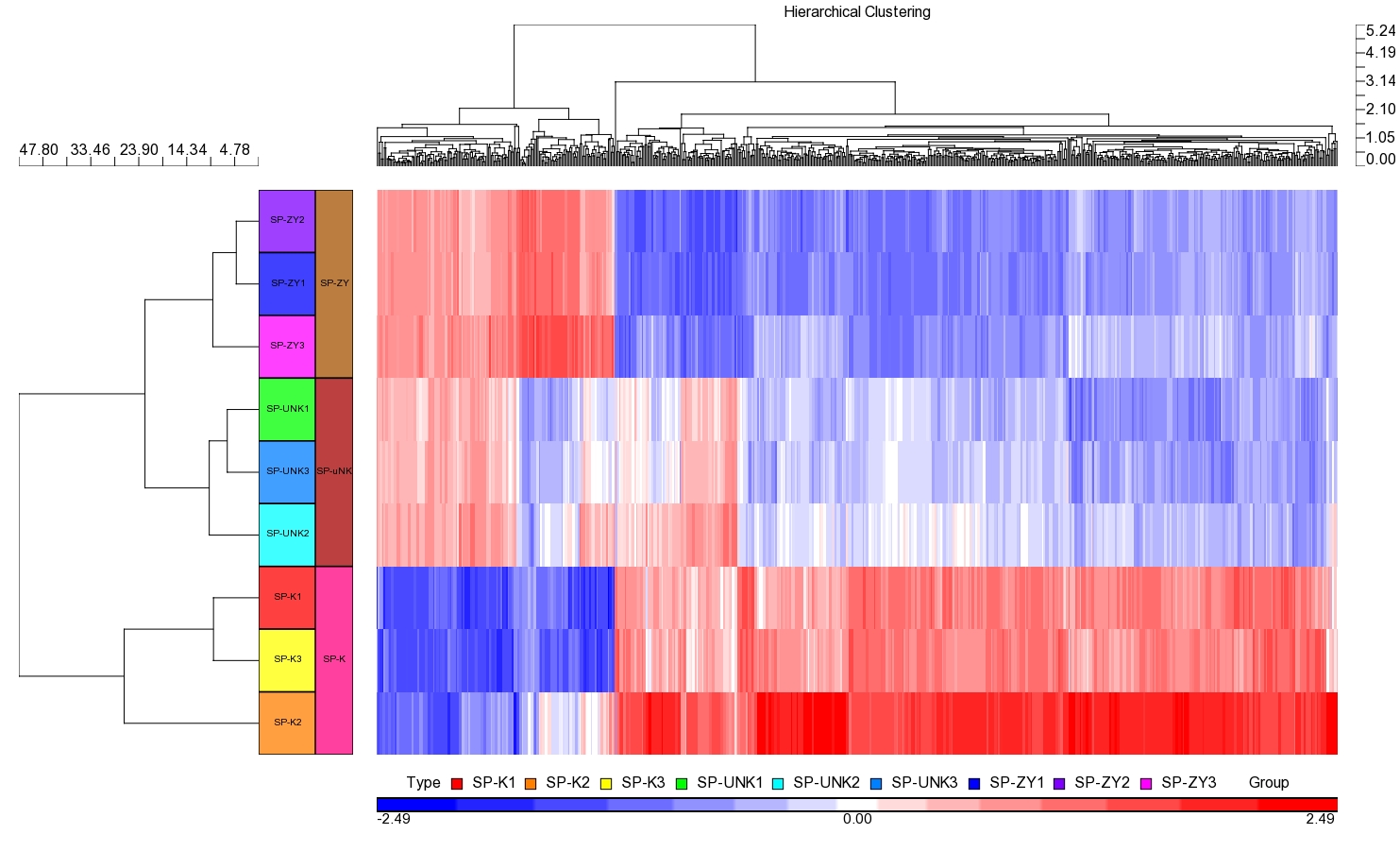

Supplement: Supplementary file 1 — Hierarchical Clustering of microarray experiments. SP-ZY represented the ZDY group; SP-uNK represented the uNK group and SP-K represented the control group. Three chips were used for each group. [file 393707.f1.jpeg]
